# Anaphylactoid purpura triggered by cellulitis as a favorable prognosis: case report and literature review

**DOI:** 10.1186/s40064-016-2792-2

**Published:** 2016-07-19

**Authors:** Natsuko Saito-Sasaki, Yu Sawada, Shun Ohmori, Daisuke Omoto, Sanehito Haruyama, Manabu Yoshioka, Daisuke Nishio, Motonobu Nakamura

**Affiliations:** Department of Dermatology, University of Occupational and Environmental Health, 1-1 Iseigaoka, Yahatanishi-ku, Kitakyushu, 807-8555 Japan

**Keywords:** Anaphylactoid purpura, Cellulitis, Inflammation

## Abstract

**Background:**

An anaphylactoid purpura affects small capillaries in the skin and other organs. Although two cases of anaphylactoid purpura exacerbated by cellulitis have been reported in Japanese literatures, its prognosis remains still unclear. Because cellulitis exacerbates various cutaneous inflammations, it has been speculated that cellulitis might also exacerbate cutaneous inflammation, such as vasculitis.

**Findings:**

In this article, we report that 78-year-old woman exhibited anaphylactoid purpura, following cellulitis. We also reviewed the literature concerning about this subject.

**Conclusions:**

This type of anaphylactoid purpura is thought to have a favorable prognosis dependent on the treatment for cellulitis.

## Introduction

An anaphylactoid purpura affects small capillaries in the skin and other organs (Jennette et al. 1994). It is known to be associated with various infections, drugs, and malignancies (González et al. 2009). Although two cases of anaphylactoid purpura exacerbated by cellulitis have been reported in Japanese literatures, its prognosis remains still unclear. Herein, we report the first case of anaphylactoid purpura triggered by cellulitis in English literature and review the literature in order to discuss the clinical course further.

## Case report

**Fig. 1:**
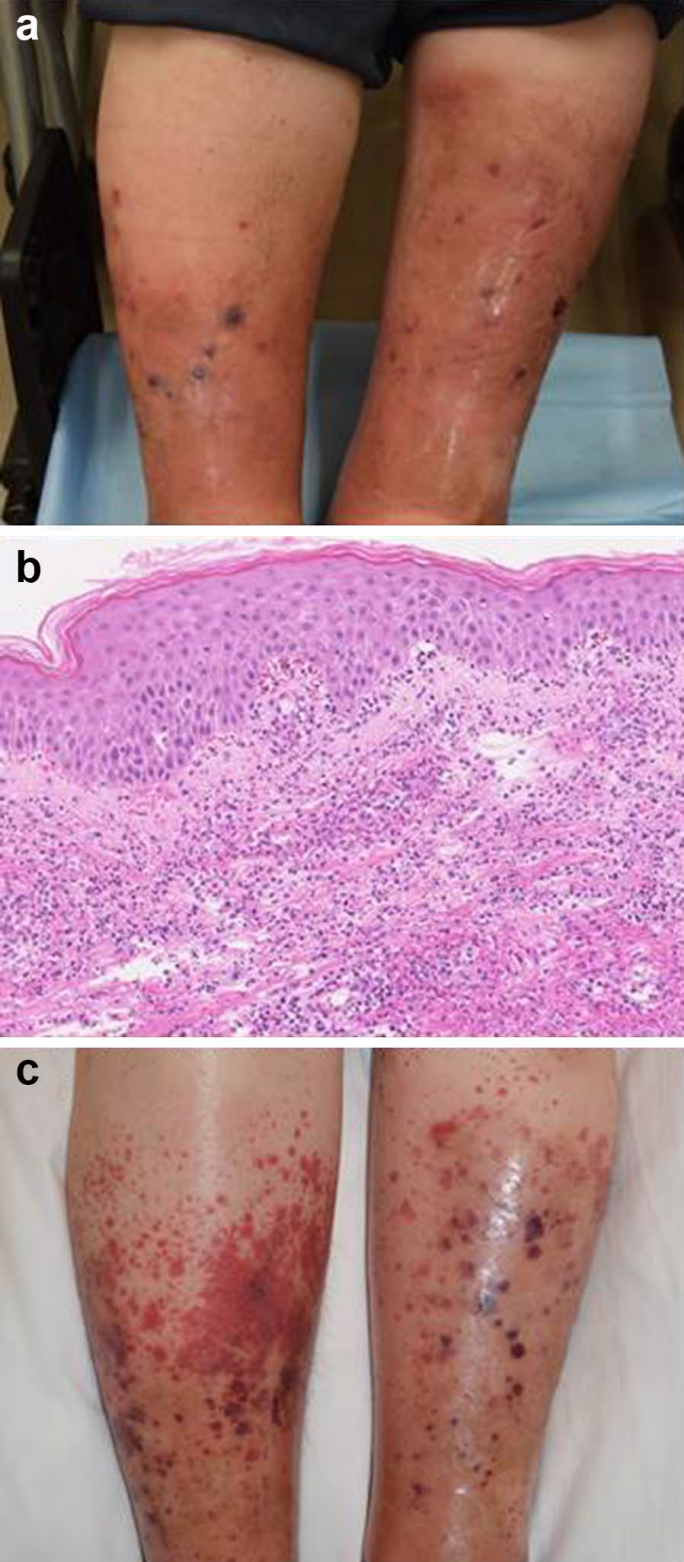
Clinical manifestation and histopathology. **a** Clinical manifestation with painful, swollen and palpable purpuric skin eruptions on both sides of her lower legs. **b** Histopathological examination exhibiting neutrophil-rich leukocytoclastic vasculitis in upper dermis with extravasation of red blood cells into dermis (hematoxylin and eosin stain; original magnification ×100). **c** Clinical manifestation of reappearance of purpura with cellulitis

A 78-year-old woman visited our hospital with a fever over 38° and for evaluation of her painful, swollen and palpable purpuric skin eruptions on her lower legs (Fig. 1a). Physical examination revealed swollen and punctate purpuric eruptions on her both lower legs. Laboratory examination showed normal leukocyte count of 8400/μl and elevated C-reactive protein level at 6.33 mg/dl (normal, <0.14 mg/dL). A skin biopsy specimen taken from a purpuric lesion on her leg revealed that neutrophil-rich leukocytoclastic vasculitis in upper dermis with extravasation of red blood cells into the dermis (Fig. 1b). Direct immunostaining shows IgA deposition surrounding small vessels in upper dermis. Based on the clinical course and laboratory examination, we diagnosed her skin eruption as an anaphylactoid purpura exacerbated by cellulitis. After the treatment with oral methyl-prednisolone 10 mg per day and cefazoline 2 g per day, her eruption improved remarkably in 2 weeks.

When cellulitis re-emerged on her lower legs, a generalized purpuric eruption reappeared on her upper arms, legs and lower abdomen (Fig. 1c). Laboratory examination showed a leukocyte count of 10,900/μl with neutrophillia (8262/μl) and elevated C-reactive at 10.77 mg/dl. Based on the clinical course and laboratory examination, we again diagnosed the eruption as anaphylactoid purpura, following cellulitis. The patient was treated with antibiotic, sulbactam/ampicillin 3 g per day and methyl-prednisolone 30 mg per day. After these treatment, her cutaneous inflammatory reaction and purpuric skin eruption rapidly subsided.

## Discussion

This case report shows a possibility that cellulitis might exacerbate an anaphylactoid purpura. We reviewed the reported two cases of an anaphylactoid purpura caused by cellulitis in Japanese literatures (Table 1). Ushigome et al. (2012) reported a case of 41-year-old male who suffered from an anaphylactoid purpura after 3 days cellulitis on his left lower leg treated with diaminodiphenylsulfone and antibiotics. Shiraishi et al. (2008) also reported a case of 78-year-old male who suffered from an anaphylactoid purpura 6 days after cellulitis on his right lower leg recovered with antibiotics. In our case, it took relatively long time (14 days) for an anaphylactoid purpura to take place following cellulitis, possibly because she was medicated by low-dose methyl-prednisolone. Furthermore, it is notable that all cases showed favorable clinical behaviors without arthralgia or renal dysfunction. Especially, the symptom of the anaphylactoid purpura was improved by mainly antibiotics treatment (Shiraishi et al. 2008). Although recurrence of cellulitis triggered anaphylactoid purpura in our case, her symptom was rapidly improved by the treatment. Good response to the treatment suggested that anaphylactoid purpura exacerbated by cellulitis has a favorable prognosis without any severe systemic symptoms, and has no require the intensive immunosuppressive therapy.

**Table 1:** Characteristics of reported cases of anaphylactoid purpura exacerbated by phlegmone

Author	Age	Sex	Location of phlegmone	Location of AP	Term AP from phlegmone (days)	Treatment for phlegmone	Treatment for AP	Reccurence
Our case	78	F	Both side of lower leg	Trunk and extremities	14	CEZ → SBT/ABPC	PSL	2nd
Ushigome et al. (2012)	41	M	Left side of lower leg	Trunk and extremities	3	CEZ	DDS	None
Shiraishi et al. (2008)	78	M	Right side of lower leg	Trunk and extremities	6	FOM	No treament	None

*CEZ* cefazolin, *SBT/ABPC* sulbactam/ampicillin, *FOM* fosfomycin, *PSL* prednisolone, *DDS* diaminodiphenyl sulfone
